# Co-Occurrence Patterns of Bacterial Communities and Resistance Genes: A Comprehensive Multi-Pen Fecal Microbiome and Resistome Study in Dairy Farms

**DOI:** 10.3390/microorganisms13112641

**Published:** 2025-11-20

**Authors:** Adriana Garzon, Rafael Portillo-Gonzalez, Gregory Habing, Bart C. Weimer, Cory Schlesener, Noelia Silva-del-Rio, Betsy M. Karle, Craig Miramontes, Richard V. Pereira

**Affiliations:** 1 Department of Population Health and Reproduction, School of Veterinary Medicine, University of California, Davis, CA 95616, USA; amgarzon@ucdavis.edu (A.G.); cschlesener@ucdavis.edu (C.S.); nsilvadelrio@ucdavis.edu (N.S.-d.-R.); ccmiramontes@ucdavis.edu (C.M.); 2 Department of Veterinary Preventive Medicine, College of Veterinary Medicine, The Ohio State University, Columbus, OH 43210, USA; portillo-gonzalez.1@osu.edu (R.P.-G.); habing.4@osu.edu (G.H.); 3 100K Pathogen Genome Project, School of Veterinary Medicine, University of California, Davis, CA 95616, USA; 4 Veterinary Medicine Teaching and Research Center, School of Veterinary Medicine, University of California, Tulare, CA 93274, USA; 5 Cooperative Extension, Division of Agriculture and Natural Resources, University of California, Orland, CA 95963, USA; bmkarle@ucanr.edu

**Keywords:** antimicrobial resistance, bovine, resistome, next-generation sequencing, metagenomics

## Abstract

Antimicrobial resistance (AMR) poses a critical public health threat, with rising multidrug resistance cases compromising treatment effectiveness. Knowledge about the resistome in dairy production systems remains limited, particularly regarding lactating cows. This study investigated the microbiome and resistome across the hospital, fresh, and mid-lactation pens on 18 conventional dairy farms in California and Ohio using shotgun metagenomic sequencing of pooled fecal samples. Pooled fecal pat samples were collected as part of a larger field study using a quasi-experimental design that assigned farms to the training intervention group (six per state) or the control group (three per state). For the training intervention group, farm worker(s), identified as having the task of diagnosing and treating adult cows on the farm, participated in a training program on antimicrobial stewardship practices. Pooled fecal samples (*n* = 7) were collected at enrollment and 3 months after the intervention was completed on each participating farm (*n* = 18). A total of 10,221 bacterial species and 345 AMR genes conferring resistance to 22 antimicrobial classes were identified. The hospital pen exhibited a higher AMR gene diversity compared to fresh and mid-lactation pens (*p* < 0.05). Several AMR genes showed bimodal distribution, suggesting complex transmission mechanisms. Network analysis revealed distinct gene correlation profiles across pens, with the hospital pen showing fewer gene interactions. Our findings suggest that farm-level antimicrobial drug use may not be the sole or primary driver of resistome composition in pooled fecal samples from dairy cattle, highlighting the need to investigate other factors influencing AMR dynamics in livestock systems.

## 1. Introduction

The increase in antimicrobial-resistant (AMR) bacteria is a significant public health concern. Given a reported increasing trend in human cases with multidrug-resistant bacteria (MDR) [1,2], infectious diseases caused by AMR bacteria have become a priority in human medicine, compromising the effectiveness of available drugs. Antimicrobial Stewardship (AMS) is a holistic approach that promotes the judicious use of antimicrobial drugs, reducing AMR to preserve antimicrobial effectiveness and availability [3]. Although AMR bacteria are monitored through surveillance programs such as the U.S. National Antimicrobial Resistance Monitoring System integrated report [1], these efforts focus on post-harvest environments and lack information that may result in the selection of AMR at the farm. Therefore, there is a knowledge gap in on-farm AMR monitoring data, which could lead to more effective efforts to reduce the selection and dissemination of AMR from cattle.

The resistome is the collection of all antimicrobial-resistance genes in pathogenic and non-pathogenic bacteria [4]. Commensal bacteria can serve as reservoirs for antimicrobial resistance determinants, which have the potential to spread to pathogenic bacteria through mobile genetic elements and therefore represent important players when considering AMR dissemination [5,6]. Currently, limited information is available about the resistome in dairy production systems, especially in lactating cows. Most resistome studies have focused on populations with intensive use of antimicrobials such as feedlots [7,8,9,10,11,12,13,14,15].

Noyes et al. [8] evaluated fecal, wastewater, and soil resistome in dairy and beef production and reported 34 different mechanisms conferring resistance to 15 classes of antimicrobial drugs. In that study, resistance mechanisms differed between beef and dairy farms, and most were related to tetracycline resistance. In addition, the diversity and composition of the resistome differed between calves and adult cattle, showing that the adult cattle resistome was less diverse than that observed in preweaned calves. They [9] also found differences between the microbiome and fecal resistome in pooled samples from feedlots from arrival until post-slaughter, identifying 42 AMR mechanisms within 17 drugs, with the most abundant being tetracyclines and the macrolide-lincosamide-streptogramin classes of antimicrobials. This study suggested that resistome changes over time may be influenced by the feeding period and antimicrobial drug use practices. Rovira et al. [15] characterized the fecal resistome of conventional and organic beef and dairy cattle production systems, finding a greater number of antimicrobial resistance genes, especially β-lactamase genes, in conventional dairy farms than in dairy farms without antibiotic exposure. According to the farm records, this research also found an association between antimicrobial resistance genes and drug classes used to treat animals.

Novel resistome data from dairy farms across different pens is an important first step to evaluate AMR genes’ relative abundance and its relation to the fecal microbiome and different herd health management practices. This study aimed to characterize pooled fecal microbiome and resistome variations across the hospital, fresh, and mid-lactation pens from dairy farms. We hypothesized that the hospital pens would exhibit higher abundances of AMR genes compared to those from fresh and mid-lactation pens. The findings of this research will enhance our knowledge of antimicrobial resistance (AMR) patterns and transmission within dairy farm environments while illuminating the relationships between bacterial communities and their associated resistance mechanisms.

## 2. Materials and Methods

All procedures were approved by The Ohio State University Institutional Review Board (#2019B047), and the study was conducted from August 2020 to March 2022.

### 2.1. Study Design

**Figure 1:**
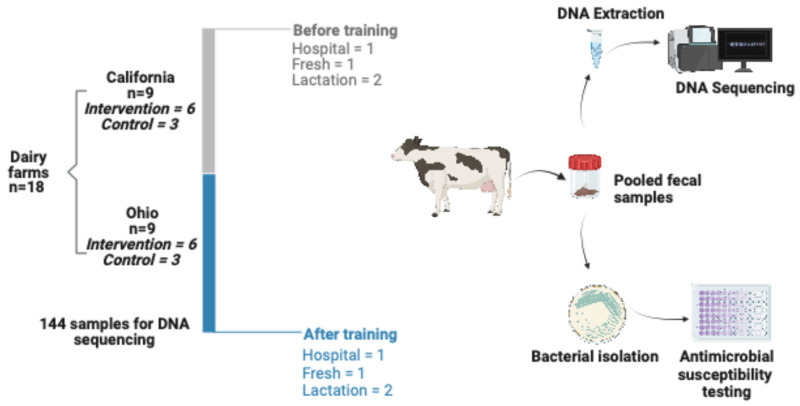
Visual outline of study design, including sampling logistics. Pooled fecal samples were collected from the hospital, fresh, and mid-lactation pens in 18 conventional dairy farms in California (n = 9) and Ohio (n = 9) and processed for shotgun sequencing. Samples were collected at two time points, enrollment and 3 months after completing the intervention on each participating farm.

Environmental pooled fecal pat samples were collected from the hospital pen (cows that have received antimicrobial treatment with milk withhold period, and representing the highest antimicrobial exposure environment and serving as a potential reservoir for AMR gene selection and amplification), the fresh pen (1 to 5 days post-partum, representing a critical transition period with heightened disease susceptibility (e.g., metritis, retained placenta) and consequently elevated antimicrobial use), and the mid-lactation pens (90 to 150 DIM, representing the baseline resistome in healthy, lactating cows with minimal antimicrobial exposure, serving as the reference population), on conventional dairies in California (*n* = 9) and Ohio (*n* = 9) (Figure 1). Fecal samples were collected as part of a larger study with a quasi-experimental design, which assigned farms to either the training intervention group (TG; six per state) or the control group (CG; three per state). For the TG, farmworker(s) identified as having the task of diagnosing and treating adult cows on the farm participated in a training program on antimicrobial stewardship practices [16]. As part of the same study, antimicrobial use (AMU) at the farm level was quantified by collecting used drug containers and manually counting them by researchers during farm visits. Treatment incidence (TI) was calculated using animal daily doses (ADD) as described previously [17]. Farms were then classified as high or low AMU based on the median TI value across all farms, where farms with TI above the median were classified as “high AMU” (*n* = 9) and those below the median as “low AMU” (*n* = 9). Importantly, this AMU classification was independent of intervention group assignment; both training intervention and control farms were represented in both high and low AMU categories. The specific TI threshold for classification was 6.75 ADD per 1000 cow-days at risk. This binary classification was used as an independent variable in statistical analyses to explore whether baseline farm-level antimicrobial use intensity was associated with resistome composition. As individual cow treatment records were not available, this farm-level AMU classification provided context for interpreting resistome patterns.

Composite fecal samples (∼200 g/sample) were collected from the ground surface of each pen by pooling feces from 10 fresh fecal pats using a 20 mL sterile sampling spoon. One composite fecal sample was collected from the hospital, one from the fresh pen, and two composite samples were collected from the mid-lactation pen. Each composite sample was placed into an 18-oz Whirl-Pak bag (Nasco, Fort Atkinson, WI, USA) and mixed by hand thoroughly. After collection, fecal samples were immediately placed on ice and transported to a laboratory for processing. Samples were aliquoted in 50 mL DNA-free tubes and frozen at −80 °C until processing.

Samples were collected twice during the study: at enrollment (time point 0) and 3 months after finishing the training program (time point 1). A total of 144 pooled fecal samples were collected for DNA extraction (Figure 1).

### 2.2. DNA Extraction and Bioinformatics

Samples were thawed first at −20 °C, followed by room temperature before DNA extraction. DNA was extracted using DNeasy^®^ PowerSoil^®^ Pro Kit (QIAGEN, Hilden, Germany), following the manufacturer’s instructions, with slight modifications. Briefly, for both wash steps, volume washes were increased to 600 µL, left on the membrane for 2 min, and centrifugated for 3 min. DNA concentration was measured at 260 nm using a NanoDrop Onec Microvolume UV-VIS spectrophotometer (Thermo Fisher Scientific, Inc., Waltham, MA, USA). Additionally, for samples with a concentration < 50 ng/μL or A260/260 < 2.0, DNA was purified using Genomic DNA Clean & Concentrator—25 (Zymo Research Corp., Tustin, CA, USA).

Library preparation was conducted in the laboratory of Dr. Bart Weimer (UC Davis, Davis, CA, USA) [18]. DNA was analyzed on the Agilent 2200 TapeStation System (Santa Clara, CA, USA) using the Genomic DNA ScreenTape assay for the integrity of gDNA. Libraries were constructed using the KAPA HyperPlus Library Preparation Kit (Roche, Indianapolis, IN, USA). Shotgun sequencing was performed using the Illumina NovaSeq S4 platform with PE150 (Illumina Inc., San Diego, CA, USA).

All raw genome sequences generated in this study are available in the NCBI SRA under BioProject accession number PRJNA186441.

Raw reads were initially trimmed with Trimmomatic (version 0.39) [18,19], and reads were assessed for quality at each step using FastQC (version 0.11.9). Reads were sorted into bovine and non-bovine reads using Kraken2 (version 2.0.8), with bovine genome reference (USDA ARS-UCD1.2; RefSeq assembly accession: GCF_002263795.1). Microbial reads were identified from non-bovine reads using the classification function in Kraken2 (version 2.1.2). Microbial reads were calculated to form phyla-, genus-, and species-level taxa abundances using Bracken (version 2.6.1).

Antibiotic resistance genes were analyzed in every genome using ARIBA (version 2.14.6). Antibiotic resistance genes were screened against the Comprehensive Antibiotic Resistance Database (CARD), the Antibiotic Resistance Gene-Annotation (ARG-ANNOT), MEGARes, ResFinder, and the National Database of Antibiotic-Resistant Organisms (NDARO). The AMR genes were classified according to resistance mechanisms and drug class using the CARD and manual curation. AMR determinants were retained for analysis if they fit the minimum criteria of 90% identity and coverage.

### 2.3. Statistical Analysis

Data analysis was conducted using RStudio (version 4.1.2). Independent variables used throughout the data analysis were sampling time points (T0, before the intervention started, and T1, 3 months after the intervention ended), states (California and Ohio), pens (hospital, fresh, and mid-lactation), and antimicrobial usage (low and high). Alpha diversity metrics were calculated at the species and gene level on a filtered phyloseq object (version 1.48.0) using the MicrobiotaProcess (version 1.16.1) package [20]. Multiple comparisons were calculated using the Wilcoxon Sum Rank Test. Center Log-ratio (CLR) was used to normalize reads via microbiome package (version 1.26.0) [21].

Beta-diversity was estimated at the species and gene level on a normalized phyloseq object, using non-metric multidimensional scaling (NMDS) ordination with Bray–Curtis dissimilarity distances. Permutational multivariate analysis of variance (PERMANOVA) based on the Bay-Curtis index with 999 iterations was used to test differences between independent variables. Unique and shared AMR genes were visualized between pens and farms with high and low AMU using the UpSet R package (version 1.4.0) [22]. To evaluate the co-occurrence of AMR genes within each pen, pairwise Spearman correlation coefficients were calculated using the cor function from the base R stats package with the method parameter set to ‘spearman’. Statistical significance was assessed using the cor.test function, and *p*-values were adjusted for multiple testing using the Benjamini–Hochberg false discovery rate (FDR) correction. Network visualization was performed using the ‘plot_network’ function from the phyloseq R package, with nodes representing individual genes and edges indicating significant co-occurrence patterns, using a Spearman coefficient |ρ| ≥ 0.75 and FDR-adjusted *p* < 0.05. Node size was scaled by gene abundance, and edge thickness was proportional to correlation strength. A pairwise probabilistic co-occurrence analysis was performed to investigate the relationships between the microbiome composition and resistome. A Spearman correlation was calculated between CLR-transformed AMR gene abundances and bacterial species abundances. Only correlations with a coefficient |ρ| ≥ 0.75 and FDR-adjusted *p* < 0.05 were considered statistically significant. The resulting correlation matrix was visualized using the corrplot package (version 0.95) [23], which generated a heatmap highlighting statistically significant associations between microbial taxa and resistance genes.

Differential abundance of species and genes between pens was performed using ANCOM-BC, with p_adj_method set to Benjamini–Hochberg [24]. Logistic regression was used to analyze genes identified as differentially abundant across samples. For each gene, two mixed-effects logistic regression models were developed. The first model examined the association between the presence of a resistance gene and the use of its corresponding antimicrobial drug class at the farm level, incorporating the farm as a random effect. The second model assessed the association between gene presence and overall farm antimicrobial usage (AMU), which was categorized as either high or low, again including farm as a random effect. For both models, potential confounding variables were tested by assessing whether their inclusion in the model affected the coefficient estimates by at least 20%, and the final models were selected based on the lowest Akaike information criterion corrected for sample size (AICC). Statistical differences were declared at *p* < 0.05 throughout all analyses unless stated otherwise.

## 3. Results

**Figure 2:**
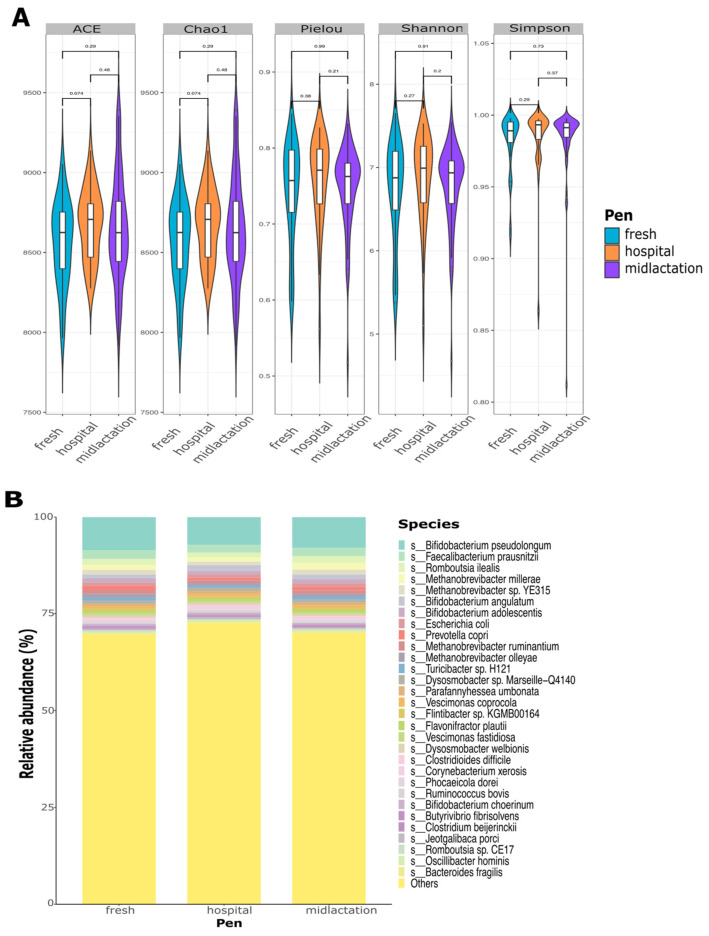
Fecal microbiome analysis of pooled samples collected from the fresh cows, hospital, and mid-lactation cow pens in 18 dairies. (**A**) Alpha diversity at the species level for ACE, Chao1, Pielou, Shannon, and Simpson diversity indexes, comparing housing pens (fresh, hospital, and mid-lactation). *p*-values represent pairwise comparisons of pens based on the Wilcoxon Sum Rank Test. *p*  <  0.05 was considered a significant difference. (**B**) Relative abundance of the top 30 bacteria species by pen group (fresh, hospital, and mid-lactation).

Microbial alpha diversity indexes at the species level did not significantly differ between pens (Figure 2A), treatment groups, or sampling time points (Supplementary Figure S1A,B). Relative abundance plots demonstrated a high diversity of bacterial composition from the pooled fecal samples, which did not differ between pens (Figure 2B), treatment groups, or sampling points (Supplementary Figure S1C,D). The top three bacterial species among the samples were *Bifidobacterium pseudolongum*, *Faecalibacterium prausnitzii*, and *Romboutsia ilealis.* The top 30 most abundant species represented only ~27% of the overall microbial abundance among all samples.

**Figure 3:**
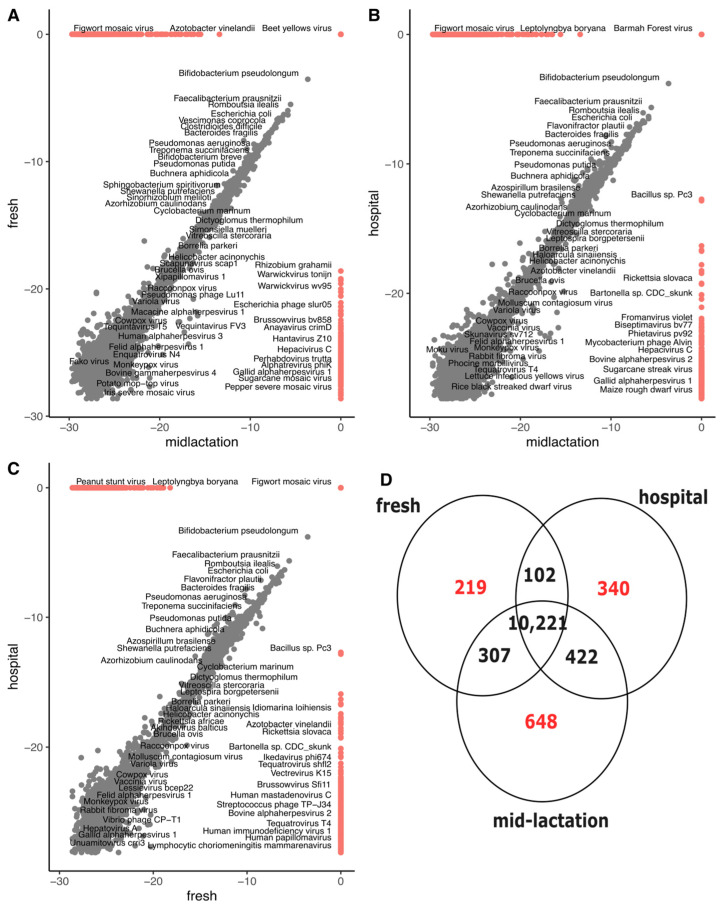
Pairwise comparison scatterplots for the log2-transformed relative abundance at the species level across cow pens in 18 dairies: (**A**) mid-lactation vs. fresh, (**B**) mid-lactation vs. hospital, (**C**) fresh vs. hospital. (**D**) Venn diagram representing the total number of bacterial species unique and shared between the three pens. Red color represents bacterial species unique to each pen, while black color represents bacterial species share between pens.

Microbial composition analysis revealed substantial diversity and distinct organism abundance patterns across different pens. We identified 10,221 bacterial species common to all three pens (hospital, fresh, and mid-lactation). The fresh pen contained 219 unique species, while the hospital pen had 340 exclusive species. The mid-lactation pen showed the highest number of unique species (n = 648) among all pens studied. Also, many viruses and bacteriophages were also identified (Figure 3). For beta diversity, three ordinations were created to evaluate differences between pens, treatment groups, and sampling points (Supplementary Figure S2A–C). PERMANOVA analysis revealed no significant differences in microbial diversity between pens, treatment groups, or sampling points.

**Figure 4:**
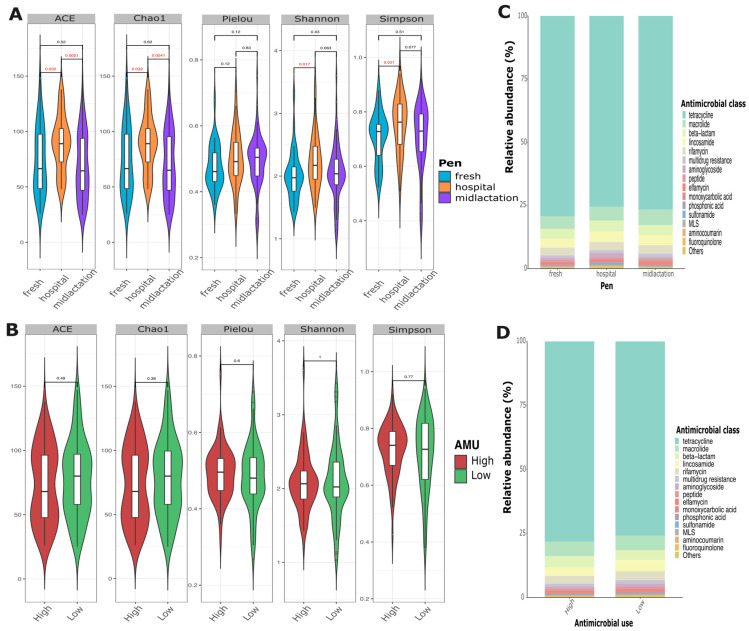
Fecal resistome analysis of pooled samples collected from the fresh cows, hospital, and mid-lactation pens. (**A**) Alpha diversity at the species level for ACE, Chao1, Pielou, Shannon, and Simpson diversity indexes, comparing housing pens (fresh, hospital, and mid-lactation) and (**B**) Antimicrobial usage level (high and low antimicrobial use at the farm). *p*-values represent pairwise comparisons of pens based on the Wilcoxon Sum Rank Test. *p*  <  0.05 was considered a significant difference (red font). Relative abundance of the top 15 antimicrobial drug classes by (**C**) Pen group (fresh, hospital, and mid-lactation), and (**D**) Antimicrobial usage level (high and low antimicrobial use at the farm).

Resistome analysis revealed high diversity in the abundance of antimicrobial resistance genes among samples. Alpha diversity indices showed that the hospital pen had a significantly higher alpha diversity compared to the mid-lactation or the fresh pen, while there was no statistical difference in diversity between the mid-lactation and fresh pens (Figure 4A). Relative abundance assessments demonstrated a high diversity of AMR gene composition from the pooled fecal samples that did not differ between pens (Figure 4C), farms with low or high AMU (Figure 4D), or individual farms (Supplementary Figure S3). The top three most common antimicrobial drug classes for which resistance was identified were tetracyclines, macrolides, and beta-lactams, with tetracycline resistance representing ~75% of the overall abundance among all samples.

**Figure 5:**
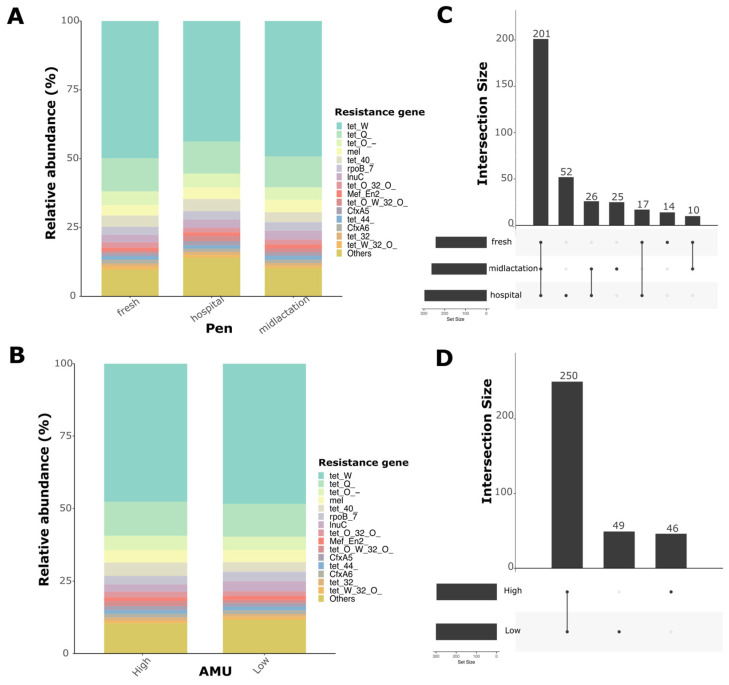
Relative abundance of the top 15 antimicrobial resistance genes by (**A**) Pen group (fresh, hospital, and mid-lactation), and (**B**) Antimicrobial usage level (high and low antimicrobial use at the farm). Upset plots for the intersection of shared and unique antimicrobial resistance genes between (**C**) Pen group (fresh, hospital, and mid-lactation), and (**D**) Antimicrobial usage level (high and low antimicrobial use at the farm).

The resistome analysis of pooled fecal samples revealed distinct gene abundance patterns across different pens. We identified 345 antimicrobial resistance genes that conferred resistance to 22 different antimicrobial drug classes. Relative abundance plots demonstrated high diversity in antimicrobial resistance genes. The three most abundant AMR genes conferred resistance to tetracycline (*tetW*, *tetQ*, *tetO*) (Figure 5A,B). Upset plots showed the number of unique and shared genes between pens and antimicrobial usage (AMU) levels (Figure 5C,D). Particularly, 201 genes were shared among pens, with the hospital pen having the highest number of unique AMR genes compared to the fresh and mid-lactation pens. For beta-diversity analysis, two ordinations were created to evaluate differences between pens and levels of antimicrobial usage at the farm (Supplementary Figure S4). PERMANOVA analysis revealed a significant difference in resistome composition between pens (*p* = 0.005), while there were no statistically significant differences between AMU levels (*p* = 0.12), despite the statistically significant difference in TI between farms categorized as low or high (median TI: 11.37 vs. 5.2 ADD per 1000 cow-days; *p* < 0.05).

**Figure 6:**
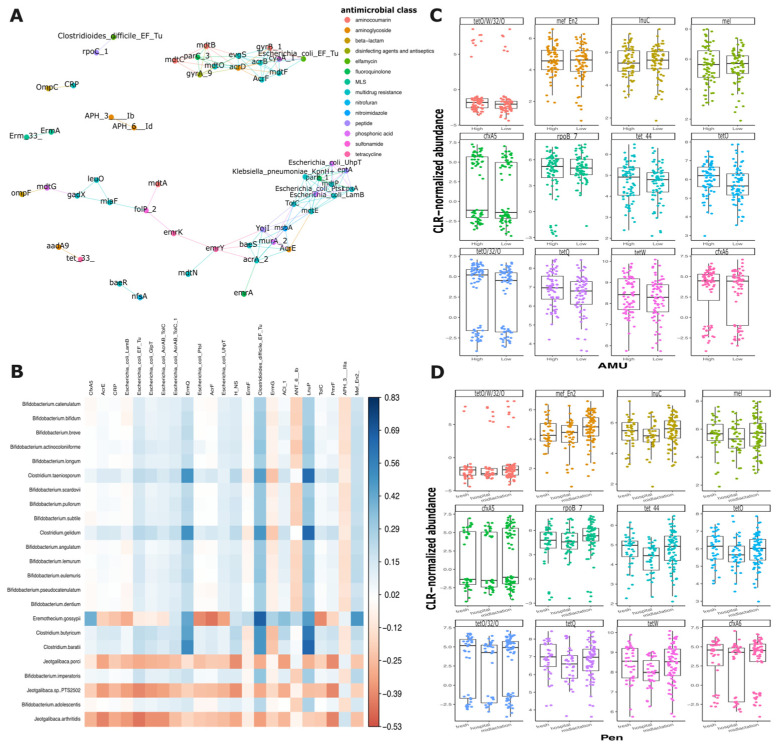
Antimicrobial resistance genes in pooled fecal samples from the hospital, fresh and mid-lactation pens. (**A**) Co-occurrence network of antimicrobial resistance genes from pooled fecal samples. Nodes representing AMR genes are color-coded by antimicrobial drug class. (**B**) Correlation plot of antimicrobial resistance genes and bacterial species. Distribution of Top 12 more common resistance genes by (**C**) Antimicrobial usage (AMU) level (high and low antimicrobial use at the farm), and (**D**) A Pen (hospital, fresh and mid-lactation).

Network analysis of antimicrobial resistance genes did not reveal significant correlations when examining the genes comprehensively or when analyzed by isolation pen (Figure 6A and Supplementary Figure S5A–C). However, multiple interactions among AMR genes from different antimicrobial classes were observed. Of particular interest, certain genes exhibited consistent co-occurrence patterns across different pens. For example, two aminoglycoside resistance genes (*aph*(*3″*)-*Ib* and *aph*(*6′*)-*Id*) and two multidrug resistance genes (*ErmA* and *Emr33*) demonstrated similar relationship networks (Figure 6A). Notably, the hospital pen exhibited the most distinct gene correlation profile, showing the lowest number of gene interactions compared to the mid-lactation and fresh pens, which displayed more comparable co-occurrence patterns. The co-occurrence analysis of AMR genes and bacterial species revealed 23 statistically significant pairwise correlations (Figure 6B). Also, two aminoglycoside resistance genes, *aph*(*3″*)-*Ib* and *aph*(*6′*)-*Id*, demonstrated strong correlations with multiple bacterial species. When analyzing the correlations by pen of origin, the hospital pen exhibited the highest number of significant correlations between AMR genes and bacterial species (n = 46), the fresh pen showed 21 significant pairwise comparisons, while the mid-lactation pen displayed the lowest number of significant correlations, with only 12 pairwise comparisons (Supplementary Figure S6A–C).

To elucidate the mechanisms of antimicrobial resistance (AMR) gene selection and persistence, we conducted a focused analysis of the top 12 most prevalent AMR genes. The genes’ distribution across farms with varying levels of antimicrobial use (AMU) and across the distinct pens (hospital, fresh, and mid-lactation) was evaluated. Surprisingly, the distribution of these genes remained consistent, showing no significant variations between farms with low and high antimicrobial usage or across different pen locations. However, several antimicrobial resistance genes (*tetO/W/32/O*, *tetO/32/0*, *cfxA5*, and *cfxA6*) exhibited a distinctive bimodal distribution pattern, suggesting complex underlying mechanisms of gene prevalence and transmission (Figure 6C,D).

**Figure 7:**
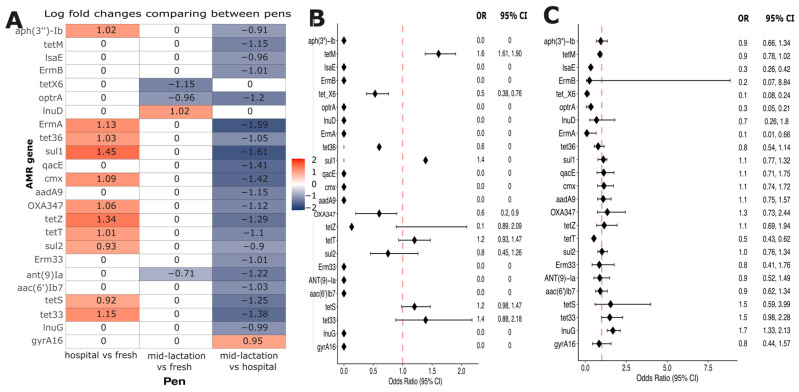
(**A**) Heatmap of natural log fold changes in abundance of the 24 antimicrobial resistance genes with an adjusted *p*  <  0.05 for pairwise comparisons between the hospital, mid-lactation, and fresh pens. Red indicates increased abundance in the comparison group; blue indicates decreased abundance in the comparison group. Zero values in white cells indicate a non-significant log-fold change in abundance. Antimicrobial exposure-adjusted logistic regression models evaluating the association between the presence of antimicrobial resistance genes differentially abundant between pens and (**B**) Farm-level use of antimicrobial drug classes associated with those resistance genes (categorized as use/no use of the specific drug class), (**C**) Overall farm antimicrobial usage level (categorized as high or low).

Analysis of Compositions of Microbiomes with Bias Correction (ANCOM-BC) was used to investigate the resistome composition variations across different pen locations. This analysis revealed 24 differentially abundant antimicrobial resistance genes. Twenty-two out of the twenty-four genes showed significantly higher abundance in the hospital pen compared to the mid-lactation pen. In contrast, one gene (*lnuD*) was more abundant in the fresh pen relative to the hospital pen, and one gene (*gyrA16*) demonstrated increased abundance in the mid-lactation pen (Figure 7A). These 24 differentially abundant genes were selected to build logistic regression models, to evaluate the association between the resistance genes and farm-level antimicrobial drug usage. Statistical modeling revealed two significant associations: first, the *tetM* gene demonstrated higher odds of occurrence (OR: 1.61, 95% CI: 1.38–1.90) in farms using tetracycline drugs to treat infectious diseases in adult cattle compared to farms without tetracycline usage (Figure 7B). Second, the *lnuG* gene exhibited higher odds (OR: 1.68, 95% CI: 1.33–2.13) in farms categorized with high antimicrobial usage relative to those with low usage (Figure 7C).

## 4. Discussion

This study used shotgun sequencing to characterize the microbiome and resistome of pooled fecal samples from adult dairy cattle fecal pats and their association with pen and farm antimicrobial use, representing one of the first of its kind in dairy cattle. The findings reveal the complex landscape of the microbiome and resistome dynamics that challenge assumptions about antimicrobial resistance transmission, selection, and persistence. The mid-lactation pen served as the healthy reference group, comprising cows in stable lactation with minimal disease incidence and low antimicrobial exposure. The fresh pen represented a transitional high-risk period, while the hospital pen contained clinically diseased animals. This design allows the comparison of resistome characteristics across a spectrum from healthy baseline (mid-lactation) through moderate risk (fresh) to high disease/treatment exposure (hospital). While individual health status was not assessed, the pen-level grouping provided a gradient of disease and antimicrobial exposure, with mid-lactation cows representing the healthy baseline population on each farm.

The microbial composition analysis demonstrated remarkable diversity, with 10,221 bacterial species common across all pens and substantial variation in unique species between different pens. Notably, the mid-lactation pen exhibited the highest number of unique species (*n* = 648), suggesting that different production stages of dairy cattle may significantly influence microbial composition. The top three bacterial species (*B. pseudolongum*, *F. prausnitzii*, and *R. ilealis*) represented only a small fraction of the overall microbial abundance, highlighting the complex and heterogeneous nature of the microbiome in cattle. Similar findings of a high microbiome diversity in cattle fecal samples have been reported, where the most abundant species or genera account for only a small percentage of the overall composition [25,26]. Multiple viruses and bacteriophages were identified as part of the microbiome. The fecal virome in cattle remains understudied and poorly understood, with only a few studies aiming to characterize the virome and with special emphasis on diseased animals [27,28]. Further research aiming to characterize and understand the virome relevance for ruminants’ gastrointestinal ecology is warranted.

The resistome analysis revealed a rich and highly diverse abundance of antimicrobial resistance genes. We identified 345 AMR genes conferring resistance to 22 different antimicrobial drug classes, with tetracycline resistance predominating at approximately 75% of overall abundance. These findings are consistent with previous studies reporting a high prevalence of tetracycline, beta-lactam, and multidrug resistance genes in cattle and dairy environments [12,29,30,31]. The hospital pen distinguished itself from other pens by having a significantly higher alpha diversity of AMR genes compared to the fresh and mid-lactation pens. This finding is consistent with expectations, as hospital pen cattle have received antimicrobial treatments for clinical diseases, creating selection pressure that may amplify pre-existing resistance genes and favor resistant bacterial populations. Cattle in hospital pens are also likely excreting both antimicrobial residues and resistant bacteria, creating an environment that selects for diverse resistance mechanisms. Intriguingly, the distribution of top AMR genes remained consistent across farms with varying levels of antimicrobial usage, challenging the assumption that increased antibiotic drug use alone has a direct effect on driving antibiotic resistance gene prevalence on a farm. However, some genes, such as *tetO/W/32/O* and *cfxA5*, exhibited a bimodal distribution, indicating complex mechanisms of gene transmission and selection. To further expand on this hypothesis, previous studies evaluating the human gastrointestinal microbiome have found similar patterns of AMR gene distribution, being attributed mainly to ecological, environmental, and host-related factors such as age group, health status, differential persistence of AMR genes, or different rates of horizontal gene transfer across microbial populations [32,33,34]. These findings suggest that the resistome may be relatively stable at the farm level compared to the individual animal level, highlighting the need for further investigation of resistome maintenance and dissemination drivers in agricultural settings.

The network analysis unveiled insights into AMR gene interactions. While comprehensive analysis did not reveal significant overall correlations, specific gene interactions were observed. Additionally, aminoglycoside resistance genes (*aph*(*3″*)*-Ib* and aph(*6′*)*-Id*) and multidrug resistance genes (*ErmA* and *Emr33*) demonstrated consistent co-occurrence patterns across different pens. The hospital pen exhibited the most distinct gene correlation profile, with the lowest number of gene interactions. To our knowledge, this represents the first study examining resistome variations across dairy farm pens, with a particular focus on hospital pen analysis. Our findings suggest that antimicrobial usage patterns at the farm level may not be a primary microbiome modifier for cattle fecal resistome, suggesting that other factors play important roles in resistome composition. While individual animal treatment records were not available for the sampled animals, this farm-level AMU classification provided context for interpreting resistome patterns. However, the lack of pen-specific or individual treatment histories limits our ability to directly link AMR gene presence to recent antimicrobial exposures at the individual level. Further research is needed to understand how microbiome composition, resistome profiles, and environmental conditions interact to develop antimicrobial resistance in livestock settings.

Our models did not identify significant associations between AMR gene occurrence and specific antimicrobial usage. The *tetM* gene was the only AMR gene that had higher odds of occurrence in farms using tetracycline for treating adult cattle infectious diseases, while the *lnuG* gene demonstrated increased odds in farms with high antimicrobial usage. Unlike the *tetM* gene, which showed specific association with tetracycline use at the farm level, *lnuG* was associated with overall AMU levels rather than specific lincosamide drug usage. This result might be a result of co-selection or indirect selection, favoring the persistence of multiple resistance genes regardless of specific drug class use or historical drug use not captured in our AMU collection. These findings underscore the nuanced relationship between antimicrobial usage and resistance gene prevalence, emphasizing the importance of longitudinal studies to understand the dynamics of antimicrobial resistance in livestock production systems. The two-timepoint sampling design (enrollment and 3 months post-intervention) might have limited our ability to capture dynamic changes in resistome composition. Antimicrobial resistance genes may fluctuate over shorter timeframes in response to antimicrobial use, seasonal changes, diet modifications, or other management factors. The lack of statistically significant differences between time points in our study may reflect the actual stability of the farm-level resistome over this period, an insufficient temporal resolution to detect transient changes, or pooling effects that masked individual animal dynamics. Longitudinal studies with more frequent sampling intervals and individual animal sampling strategies might be able to further characterize temporal resistome dynamics and identify factors driving short-term fluctuations in AMR gene prevalence.

A limitation of this study is the use of pooled fecal samples, which prevents assessment of individual animal variation in microbiome and resistome composition. While this approach does not allow us to investigate individual-level dynamics and potentially underestimates the true diversity of AMR genes present on farms, it was selected as our overarching goal was a farm-level resistome characterization. Our primary objective was to characterize the overall resistome profile at the pen and farm level rather than individual animal carriage, making pooled samples appropriate for detecting prevalent resistance genes within each management group. Pooled fecal samples better represent the resistome to which all animals in a pen are exposed, which is relevant for understanding potential transmission dynamics and environmental contamination. However, we recognize that this approach is not suitable for assessing within-pen heterogeneity or determining whether rare AMR genes are broadly distributed or concentrated in a few individuals. Future studies should incorporate individual-level sampling to complement farm-level characterization and better understand AMR gene distribution patterns among individuals within pens.

Future research should focus on longitudinal studies tracking AMR gene dynamics, investigating the mechanisms driving bimodal gene distribution patterns, and exploring the ecological factors influencing microbial and resistome diversity.

## 5. Conclusions

Our research highlights the complex and dynamic nature of microbial and antimicrobial resistance ecosystems in dairy cattle production environments. Our findings indicate that while antimicrobial use undoubtedly plays a role in AMR gene selection (as evidenced by the *tetM*–tetracycline association), farm-level antimicrobial use was not the dominant determinant of overall resistome composition in pooled fecal samples. Resistome patterns showed stronger associations with pen type (*p* = 0.005) than with AMU level (*p* = 0.12), suggesting that factors associated with pen environment, including animal health status, stress levels, microbial community stability, management practices, and environmental conditions, may collectively exert stronger influences on resistome composition than antimicrobial use alone. This finding emphasizes the multifactorial nature of AMR ecology and the importance of considering diverse management and environmental factors, beyond antimicrobial use reduction, in comprehensive antimicrobial stewardship strategies. An improved understanding of the interactions between management practices, microbiome composition, and resistome dynamics will be crucial for developing effective strategies to mitigate the spread of antimicrobial resistance in livestock production systems.

## Data Availability

All raw genome sequences generated in this study are available at The 100K Pathogen Genome Project BioProject (NCBI PRJNA186441) under BioProject accession number PRJNA1245445.

## Supplementary Materials

The following supporting information can be downloaded at: https://www.mdpi.com/article/10.3390/microorganisms13112641/s1, Figure S1: Fecal microbiome analysis of pooled samples collected from the fresh cows, hospital, and mid-lactation pens. Alpha diversity at the species level comparing (A). Treatment groups (control, in-tervention) (B). Sampling time points (Before starting the intervention, T1, 6 months after complet-ing the intervention, T2). *P*-values represent pairwise comparisons based on the Wilcoxon Sum Rank Test. *P* < 0.05 was considered a significant difference. Relative abundance of the top 30 bac-teria species by (C). Treatment groups (control, intervention), (D). Sampling time points (Before starting the intervention, T1, 6 months after completing the intervention, T2).; Figure S2: Nonmetric multidimensional scaling (NMDS) based on Bray-Curtis dissimilarity of the center log ratio normalized species-level read counts of pooled fecal samples microbiome for (A). Pen (fresh, hospital and mid-lactation), (B). Treatment group (Intervention and control), and (C). Sampling time points (Before starting the intervention, T1, 6 months after completing the intervention, T2). Ellipses correspond to 95% confidence interval. *P*-values represent PERMANOVA analysis. *P* < 0.05 was considered a significant difference; Figure S3: Relative abundance of the top 15 (A). Antimicrobial drug classes and (B). Antimicrobial genes presented on pooled fecal samples from dairy cattle stratified by farm of origin. Eighteen farms were included in the study. Farms 1–9 belong to the state of California and 10–18 to the state of Ohio.; Figure S4: Principal coordinate analysis (PCoA) based on Bray-Curtis dissimilarity matrix of the center log ratio normalized genes read counts of pooled fecal samples for (A). Pen (fresh, hospital and mid-lactation), (B). Antimicrobial usage level (high and low antimicrobial use at the farm). Ellipses cor-respond to 95% confidence interval. *P*-values represent PERMANOVA analysis. *P* < 0.05 was con-sidered a significant difference; Figure S5: Co-occurrence network of antimicrobial resistance genes from pooled fecal samples. Nodes repre-senting AMR genes are color coded by antimicrobial drug class, for (A). mid-lactation, (B). hospital, and (C). fresh cow pens; Figure S6: Correlation plot of antimicrobial resistance genes and bacteria species by (A). Mid-lactation, (B). Fresh, and (C). Hospital pens; Table S1: Metadata for the shotgun sequencing of pooled fecal samples (*n* = 144) from dairy cattle included in the study; Table S2: Metadata for farm allocation and antimicrobial use.
