# Supplementary material for: Co-Occurrence Patterns of Bacterial Communities and Resistance Genes: A Comprehensive Multi-Pen Fecal Microbiome and Resistome Study in Dairy Farms
Source: Microorganisms. 2025 Nov 20;13(11):2641. doi: 10.3390/microorganisms13112641 (PMC12654601; doi:10.3390/microorganisms13112641)
Supplement: Supplementary file 1 [file microorganisms-13-02641-s001.zip › Supplementary Figures.pdf]

Supplementary Materials

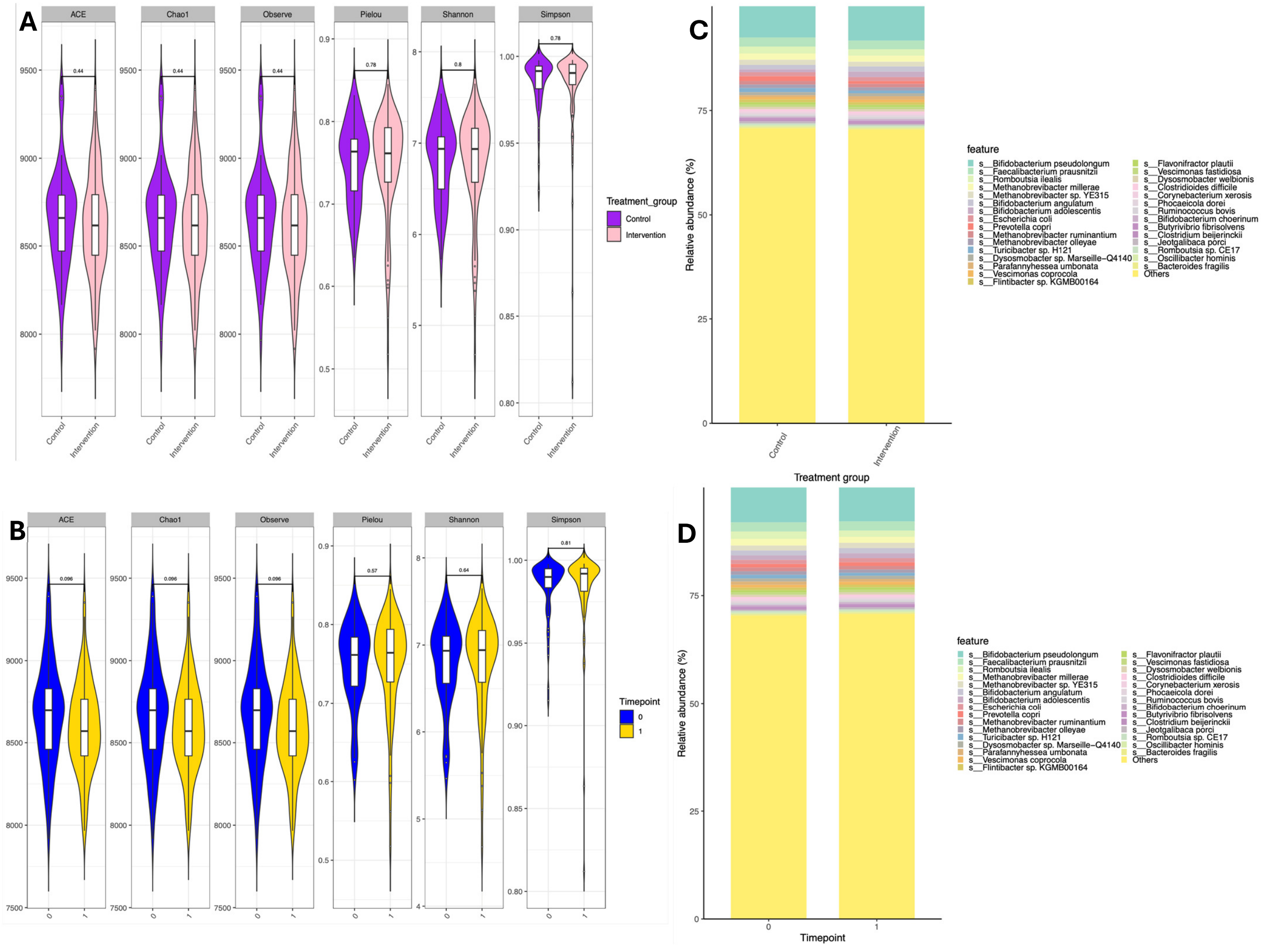

Supplementary Figure S1. Fecal microbiome analysis of pooled samples collected from the fresh cows, hospital, and mid-lactation pens. Alpha diversity at the species level comparing (**A**). Treatment groups (control, intervention) (**B**). Sampling time points (Before starting the intervention, T1, 6 months after completing the intervention, T2). *P*-values represent pairwise comparisons based on the Wilcoxon Sum Rank Test. $P < 0.05$ was considered a significant difference. Relative abundance of the top 30 bacteria species by (**C**). Treatment groups (control, intervention), (**D**). Sampling time points (Before starting the intervention, T1, 6 months after completing the intervention, T2).

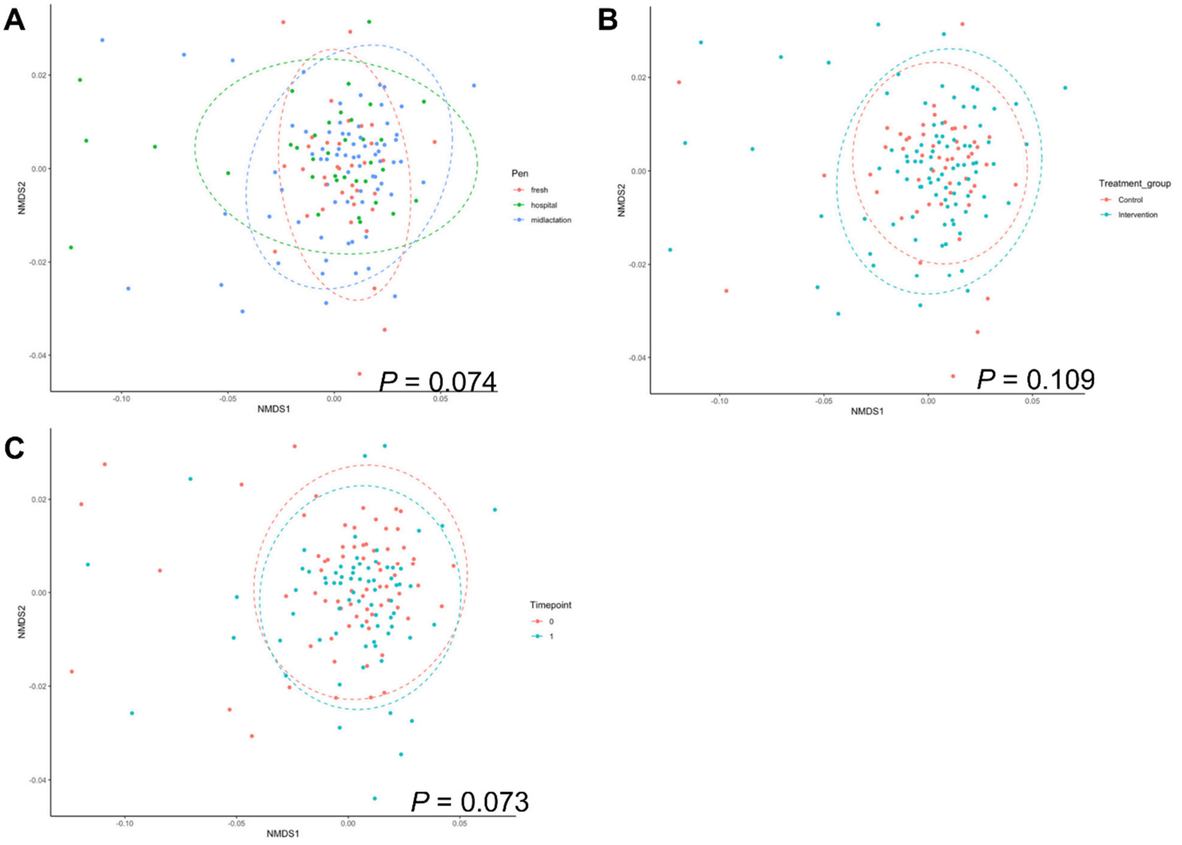

Supplementary Figure S2. Nonmetric multidimensional scaling (NMDS) based on Bray-Curtis dissimilarity of the center log ratio normalized species-level read counts of pooled fecal samples microbiome for (**A**). Pen (fresh, hospital and mid-lactation), (**B**). Treatment group (Intervention and control), and (**C**). Sampling time points (Before starting the intervention, T1, 6 months after completing the intervention, T2). Ellipses correspond to 95% confidence interval. P-values represent PERMANOVA analysis. $P < 0.05$ was considered a significant difference.

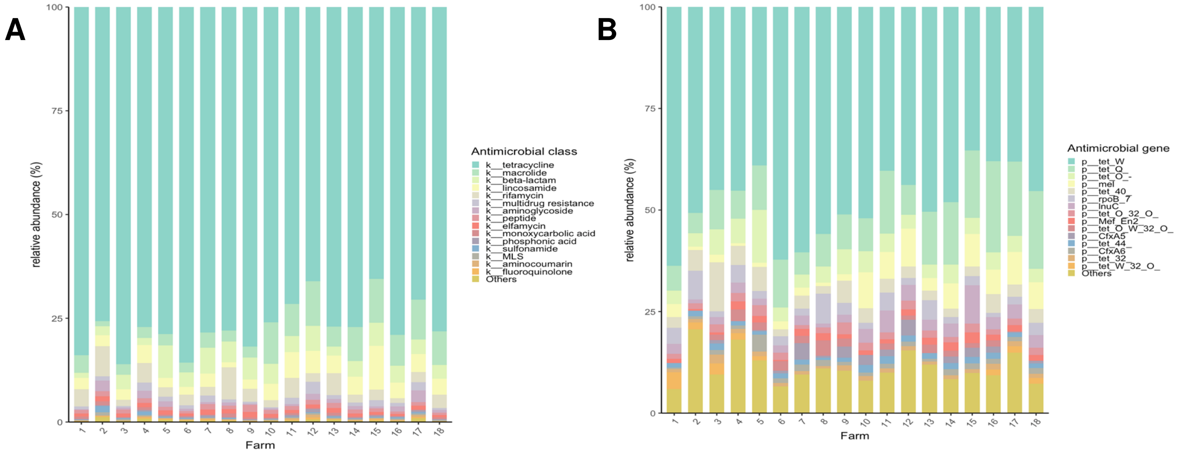

Supplementary Figure S3. Relative abundance of the top 15 (**A**). Antimicrobial drug classes and (**B**). Antimicrobial genes presented on pooled fecal samples from dairy cattle stratified by farm of origin. Eighteen farms were included in the study. Farms 1-9 belong to the state of California and 10-18 to the state of Ohio.

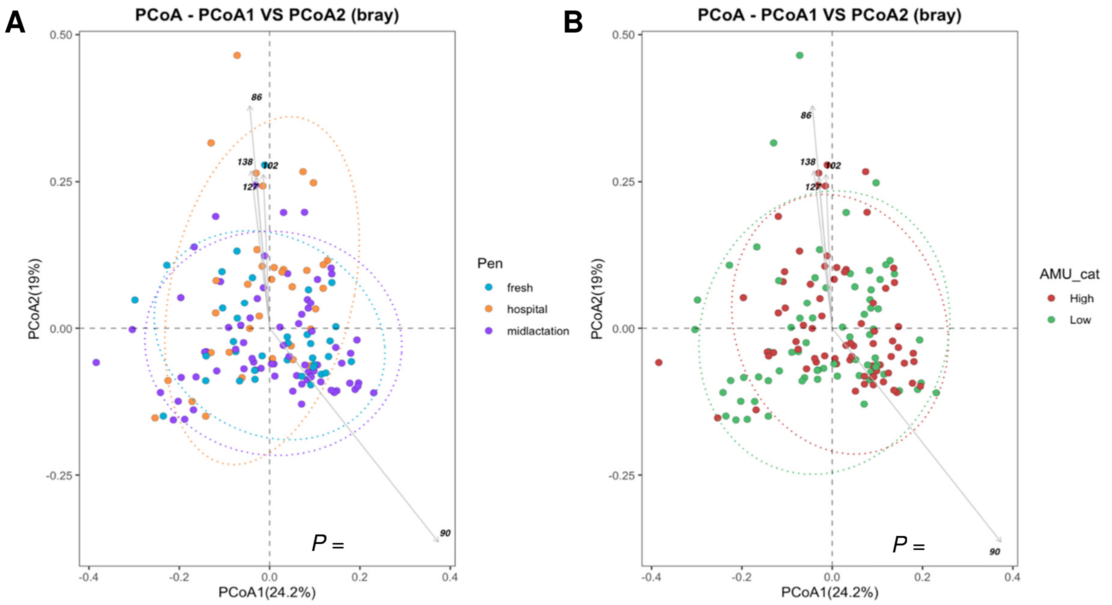

Supplementary Figure S4. Principal coordinate analysis (PCoA) based on Bray-Curtis dissimilarity matrix of the center log ratio normalized genes read counts of pooled fecal samples for (**A**). Pen (fresh, hospital and mid-lactation), (**B**). Antimicrobial usage level (high and low antimicrobial use at the farm). Ellipses correspond to 95% confidence interval. P-values represent PERMANOVA analysis. $P < 0.05$ was considered a significant difference.

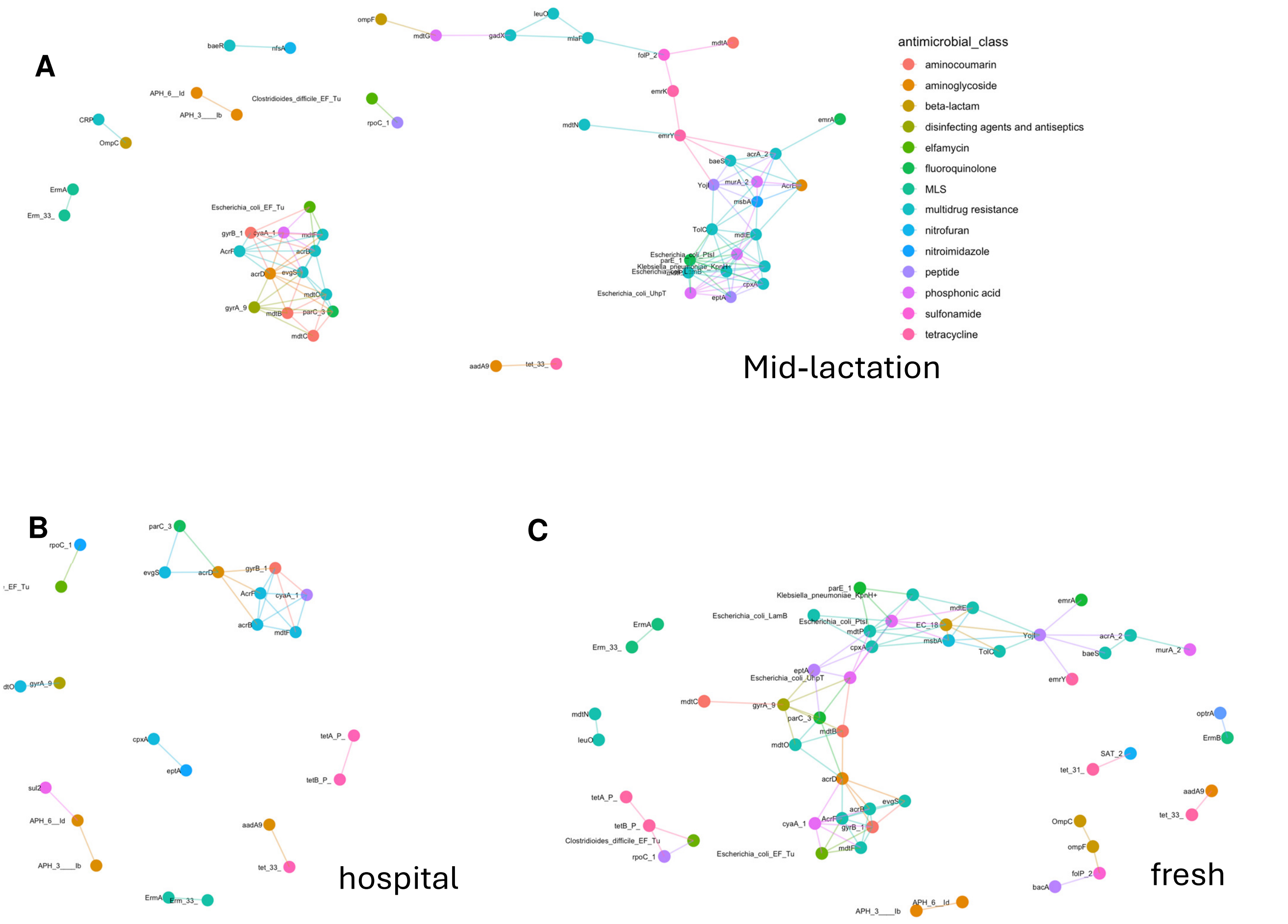

Supplementary Figure S5. Co-occurrence network of antimicrobial resistance genes from pooled fecal samples. Nodes representing AMR genes are color coded by antimicrobial drug class, for (**A**). mid-lactation, (**B**). hospital, and (**C**). fresh cow pens.

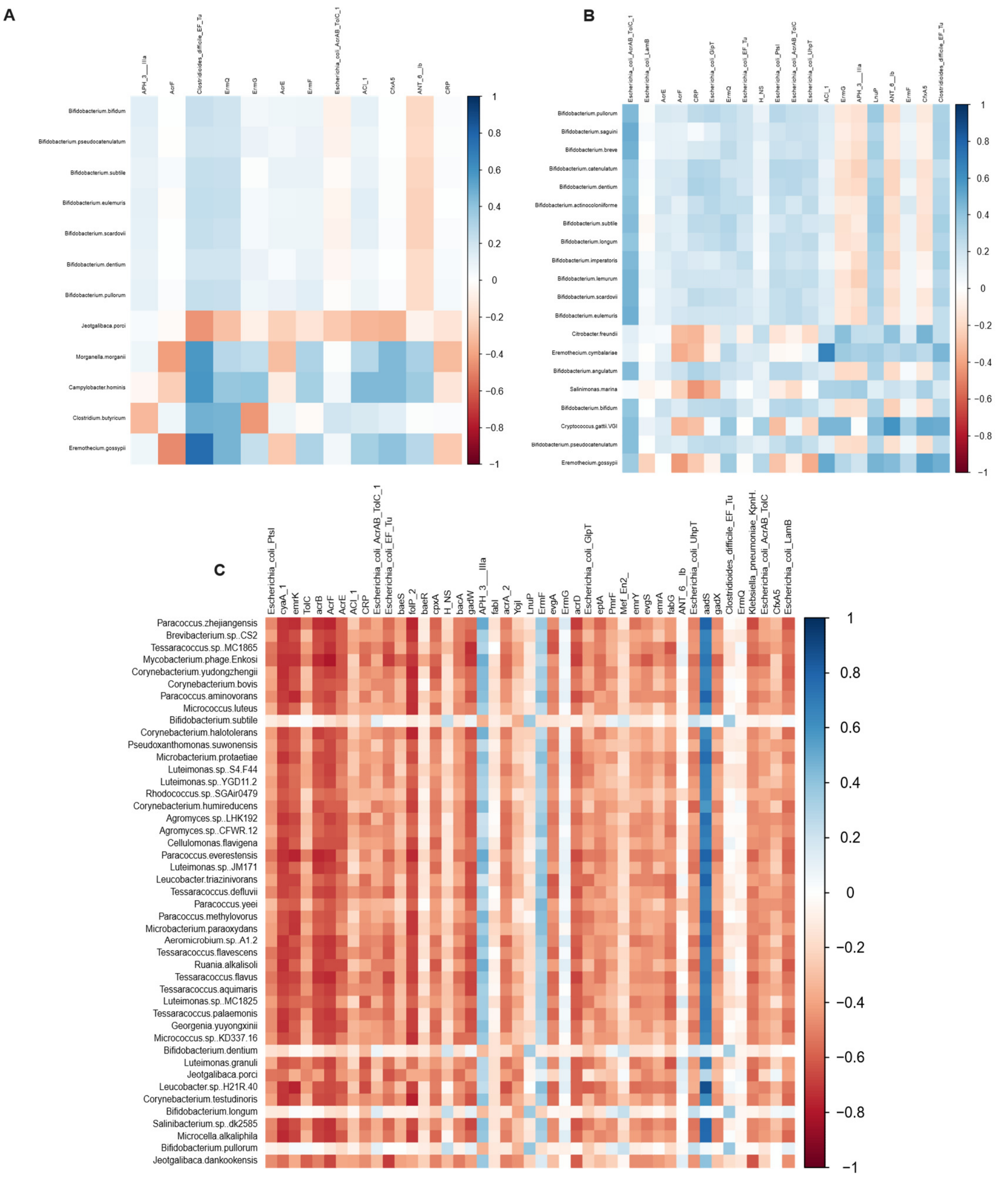

Supplementary Figure S6. Correlation plot of antimicrobial resistance genes and bacteria species by (**A**). Mid-lactation, (**B**). Fresh, and (**C**). Hospital pens.
